# The Third Pillar of Precision Medicine — Precision Delivery

**DOI:** 10.1002/mco2.70200

**Published:** 2025-04-28

**Authors:** Avnesh S. Thakor

**Affiliations:** ^1^ Department of Radiology Center for Interventional Radiology Innovation at Stanford (IRIS) School of Medicine Stanford University Palo Alto California USA

**Keywords:** cellular interactions, locoregional delivery, microenvironment modulation, precision delivery, precision medicine, targeted delivery

## Abstract

Precision Medicine is thought of as having two main pillars: Precision Diagnosis and Precision Therapy. However, for Precision Medicine to reach its full potential, a third pillar is needed that we propose to call *Precision Delivery*. In the laboratory, many therapies show great efficacy when tested directly with target cells. However, upon clinical translation, they are often given via intravenous or oral administration, resulting in their systemic distribution. To ensure therapies reach target sites at the correct therapeutic levels, they are often given at higher concentrations. However, this can be associated with off‐target effects, side‐effects, and unwanted interactions. Delivery strategies can help mitigate this by “spatially re‐coupling” therapies in vivo with target cells. This review explains the concept of *Precision Delivery*, which can be thought of as three interconnected, but independent, modules: targeted delivery, microenvironment modulation, and cellular interactions. While locoregional approaches directly deliver therapies into target tissues through endovascular, endoluminal, percutaneous, and implantation techniques, microenvironment modulation technologies facilitate the movement of therapies across biological barriers and through tissue matrices, so optimized therapies can reach and interact with target cells. We highlight new innovations driving advances in *Precision Delivery*, while also discussing the considerations and challenges that *Precision Delivery* faces as it becomes increasingly integrated into treatment workflows.

## Graphical abstract

For Precision Medicine to reach its full potential, a third pillar called *Precision Delivery* is starting to evolve to complement the existing two pillars of *Precision Diagnosis* and *Precision Therapy. Precision Delivery* will help ensure the right therapy for the right disease will be able to reach the right location at the right time

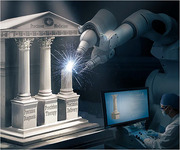

## 1 Introduction

**FIGURE 1:**
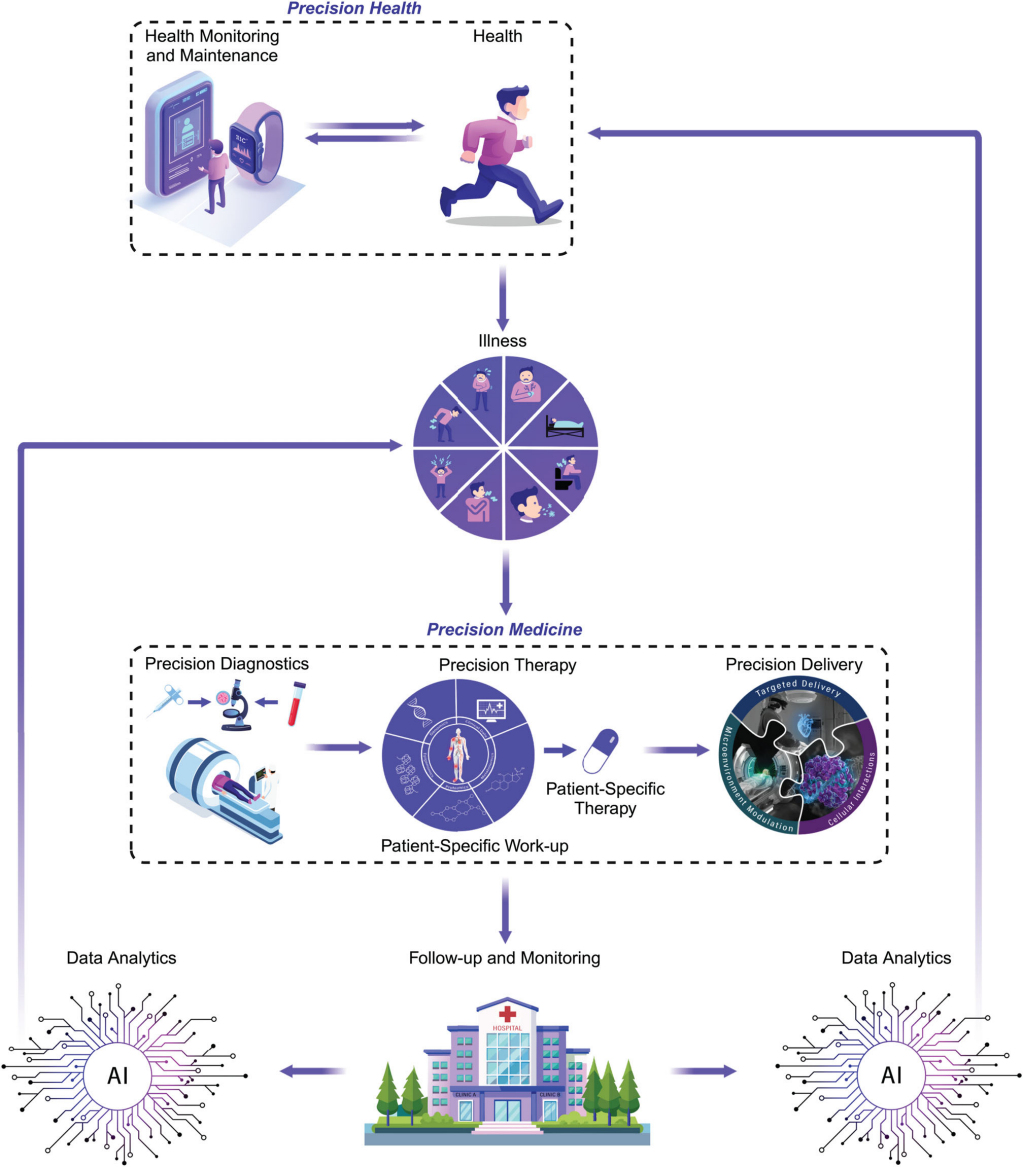
Overview of precision health and precision medicine for delivering personalized medicine. Precision Health helps to monitor and maintain an individual in a state of health and wellbeing by promoting disease prevention behaviors and detecting disease earlier using health‐monitoring devices and technologies that collect actionable data. If patients become ill, they enter Precision Medicine which encompasses: *Precision Diagnosis* (which detects, identifies and characterizes the disease process that is making a patient feel ill), *Precision Therapy* (which identifies the correct therapy to treat specific pathways driving the disease), and *Precision Delivery* (which helps ensure the correct therapy reaches target cells contributing to the disease). *Precision Delivery* has three independent modules—targeted delivery, microenvironment modulation, and cellular interactions, all of which need to be considered and optimized to ensure optimal therapy delivery in patients. After treatment, patients are followed up; if the therapy was successful in treating the disease, they re‐enter Precision Health, but if the disease persists or is redetected, they again re‐enter Precision Medicine. Data collected as part of health maintenance, disease monitoring, and subsequent therapeutic interventions for each patient will then be processed using artificial intelligence (AI) models to assess optimal and sub‐optimal responses; this will provide continuous and iterative feedback to help guide both Precision Health and Precision Medicine.

While Precision Health helps to monitor and maintain an individual in a state of health and wellbeing [1], Precision Medicine aims to restore the health of an individual when they enter a state of illness [2] (Figure 1). Traditionally, Precision Medicine is thought of as having two pillars: *Precision Diagnosis*—which characterizes diseases using genetic testing, molecular profiling, and advanced imaging; and *Precision Therapy*—which creates personalized treatments based on molecular drivers of a disease, as well as the unique genetic makeup of an individual.

Although new therapies show significant potential in the laboratory when they are “spatially coupled” with target cells in cell culture and/or array testing, a significant proportion ultimately fail to make it through United States Food and Drug Administration (US FDA) approval, in what is commonly referred to as the “valley of death” [3]. In many cases, this is due to variable and sub‐therapeutic responses during clinical trials, attributed, in large part, to how these therapies are translated and given to patients. Conventionally, this occurs via intravenous (IV) or oral (PO) administration, resulting in their systemic distribution, which when combined with first pass metabolism and/or organ entrapment, results in very little therapy reaching target cells (i.e., they are now “spatially uncoupled” with target cells). One solution is to administer therapies at higher concentrations; however, this is often associated with significant issues including off‐target effects, side‐effects, and unwanted interactions.

To keep therapies at lower concentrations to mitigate these adverse effects, while concurrently ensuring they reach therapeutic levels at a site of interest, delivery strategies can be used to “spatially re‐couple” therapies in vivo with target cells. For certain therapies, simple delivery techniques have already been adopted, as demonstrated by drug inhalation for airway diseases, drug injection into affected joints, and topical cream application for skin disorders. Nevertheless, advances in (bio)engineering, polymer science, imaging, and additive manufacturing have aided in the development of more specialized medical devices and technologies that can now target, and modulate, almost any part of the human body with exquisite precision. Accordingly, we are now seeing an increasing adoption of more complex targeted delivery approaches for a wide range of diseases. In oncology, there has been a significant expansion in the use of locoregional techniques for curative therapy, local disease control, or even to help transition to surgery. One example is intraarterial (IA) therapy to treat liver tumors, with multiple large randomized clinical trials showing that it improves therapy efficacy, reduces side effects, improves quality of life, and importantly, increases survival benefit when compared with systemic IV therapy [4, 5]. While promising results have also been seen using IA chemotherapy in other cancers (i.e., melanoma [6], pancreatic [7], urological—kidney and bladder [8], head and neck tumors [9], retinoblastoma [10], cholangiocarcinoma [11]), definitive conclusions still require larger trials. Interestingly, for brain tumors (i.e., glioblastoma), the benefits of IA chemotherapy were initially shown to be limited, especially when examining overall survival compared to conventional IV administration [12]. However, when IA chemotherapy is combined with mannitol, which opens the blood–brain barrier (BBB), data are more encouraging and show improved survival benefits [12]. Hence, it is transpiring that delivery is more complex than initially thought, going beyond just the physical delivery of therapies into organs, but needing to also consider the use of adjunctive drugs and technologies to overcome physiological barriers (i.e., BBB, endothelial–epithelial interfaces, stromal/fibrous tissue causing increased extracellular matrix [ECM]) or employing strategies to avoid local therapy inactivation (i.e., hostile local microenvironments, drug efflux pumps) [13].

Hence, for Precision Medicine to realize its full potential, a third pillar is needed that we propose to call: *Precision Delivery*, which can be considered as a framework of three interconnected modules: (i) *targeted delivery* (i.e., direct delivery of therapies to specific sites); (ii) *microenvironment modulation* (i.e., facilitating movement of therapies through complex tissue matrices and barriers to allow them to reach target cells); and (iii) *cellular interactions* (i.e., optimizing how therapies interact with target cells to initiate their desired effect(s)) (Figure 1).

## 2 The Modules of *Precision Delivery*

### 2.1 Module 1 — Targeted Delivery

**FIGURE 2:**
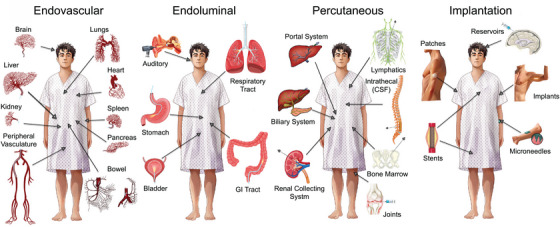
Main categories for targeted delivery. One module of *Precision Delivery* is: Targeted delivery. This describes how different organs in the human body can be accessed and targeted using four main categories: (i) endovascular (i.e., through blood vessels using devices like catheters), (ii) endoluminal (i.e., through organs which have cavities or are hollow using devices like optical scopes), (iii) percutaneous (i.e., through the skin using devices like needles), and (iv) implantation (i.e., through placement of physical structures inside or onto the human body) approaches.

The goal of targeted delivery is to avoid loss of therapy that can occur following conventional PO administration (i.e., destruction from the high acidity of the stomach or inactivation by the liver following gastrointestinal absorption) or IV administration (i.e., loss of larger therapies, like cells, due to lung entrapment; or loss of smaller therapies, especially micro/nanomedicines, from sequestration by the reticuloendothelial system and kidneys). To overcome these issues, four distinct categories have emerged to directly deliver therapies to target organs: (i) *Endovascular approaches*: these use imaging to guide catheters into different organs via their blood supply; (ii) *Endoluminal approaches*: these use fiber optic technologies in the form of endoscopes to visualize, and access, luminal canals/cavities; (iii) *Percutaneous approaches*: these use imaging to guide needles or minimally invasive technologies through the skin to deeper locations in the body; (iv) *Implantation approaches*: these use direct application of devices onto the skin, or surgical techniques to create artificial spaces that can accommodate devices in deeper anatomical locations (Figure 2).

Over the years, the technologies within these categories have developed increasing levels of sophistication to not only enable organs to be targeted with increasing precision [14, 15], but also enable delivery of advanced therapeutics. For endovascular approaches, several core technologies have served as platforms for the development of new medical devices. For example: (i) balloons have evolved to have flanges that can cut through fibrous scar tissue (cutting balloons), transferable therapy coatings (drug eluting balloons), or contain technologies that can deposit energy (radiofrequency ablation [RFA] balloons); (ii) stents have evolved to contain occlusive fabrics (stent grafts), redirect blood flow (flow diverting stents), deliver therapies in a controlled manner over time (drug eluting stents), or dissolve over time (biodegradable stents); (iii) injectable materials have evolved to act as vehicles for controlled therapy release (drug eluting spheres/particles), physical occlusion (detachable coils and vascular plugs), or have fine‐tuned polymerization characteristics for controlled embolization (glue and onyx); and (iv) devices can now be placed inside vessels to remove blood clots (thrombectomy devices), replace defective structures (transcatheter valve replacement), deliver energies for tissue destruction (laser, cryoablation, and RFA catheters), or bypass barriers for therapy delivery (new emerging microneedle balloon catheters that can penetrate the vessel wall). Interestingly, positive‐feedback loops then organically develop between different technological advances and novel therapies. For example, the development of high‐resolution imaging equipment and small diameter catheters, that even have steerable tips, have allowed for super‐selective access to almost any location in the human body. Accordingly, this catalyzed the development of highly potent catheter‐based therapies (i.e., Yttrium‐90 radiation spheres for tumor treatment), which could only be created once delivery to very specific locations could be ensured, given the potential of these therapies to induce serious adverse effects if delivered to non‐target locations [16].

Similar technological advances can also be seen with endoscopes (which have evolved into powerful luminal delivery tools given their improved flexibility and light sources, coupled with their ability to incorporate therapeutic channels for drug and device delivery), as well as needles (which have evolved from a basic sampling tool, to a high‐tech instrument that can deliver therapies either directly into tissues via hollow‐bores, or deposit thermal energy into tissues via solid bores that can be used as a probe or antenna). Biotechnological advances have also been instrumental in creating implantable delivery technologies that can be placed superficially (i.e., microneedle patches for transdermal delivery of therapies like DNA vaccines [17], insulin [18], or drugs [19]), underneath the skin within the subcutaneous or intramuscular space (i.e., bioscaffolds that can accommodate cellular therapies that function as an “artificial organ” [20, 21]), or deep inside the body (i.e., bioabsorbable meshes that can provide strength to tissues, while also releasing bioactive compounds to promote tissue regeneration [22]).

### 2.2 Module 2 — Microenvironment Modulation

Following delivery, therapies need to overcome several tissue barriers to reach target cells, starting with cellular interfaces (i.e., endothelial cells for those delivered in the blood stream and epithelial cells for those delivered into biological lumens) to tissue matrices that consist of the ECM (composed of collagen and glycoproteins) and interstitial space (IS; regulated by the lymphatic system). Appreciation of these barriers is essential, as select organs have specialized barriers (i.e., brain with the BBB, kidney with glomeruli, and fetus with the placenta) and diseases can often alter barrier function (i.e., some tumors have increased stroma, ECM and high interstitial pressures that limit therapy access, while other inflammatory diseases have dysfunctional/leaky barriers making therapy retention difficult) [23, 24, 25].

For decades, we have known that modulating tissue microenvironments can affect these barriers to help therapy delivery; initial strategies included manipulating local physiological parameters (i.e., pH, osmolarity, temperature, and oxygenation) or using specific drugs/antibodies to change tissue receptors to sensitize tissues. However, these approaches have challenges in being able to localize these effects at specific anatomical locations. Accordingly, this led to the development of innovative technologies that can now spatially and temporally modulate tissue microenvironments. This is best illustrated for BBB opening where hyperosmolar agents, like mannitol, were initially used but given they affected the entire BBB, they were associated with side effects, such as headaches, in addition to exposing the whole brain to circulating pathogens and toxic materials [26, 27]. Several technologies were then developed to open the BBB at specific locations, with focused ultrasound (FUS) emerging as a leading approach given it can temporarily and noninvasively open the BBB at both superficial and deep locations, especially with the concomitant use of microbubbles [28, 29]. Clinical trials are now underway testing FUS for BBB opening to augment therapy delivery to tumors and neurodegenerative disorders [30].

In other tissues, innovative technologies like iontophoresis are being explored, which uses low‐voltage continuous electrical currents to change the charge or polarity of tissues to facilitate therapy delivery. Initially this was used to promote transdermal delivery of drugs [31], with newer indications including enhancing chemotherapy delivery into solid tumors that have penetration challenges [32]. Electroporation is another microenvironment modulating technology, which uses high voltage pulsed electrical fields that can transiently destabilize cell membranes allowing the entry of macromolecules, nucleic acids, and gene therapies into cells. While this technology is readily used in vitro and ex vivo (i.e., engineering chimeric antigen receptor‐T cells) [33], its is in vivo use has been more challenging, but is being explored as part of electrochemotherapy, gene electrotransfer, and electrovaccines, especially in superficial tissues [34, 35].

### 2.3 Module 3 — Cellular Interactions

Once therapies reach target cells, they interact either via: (i) membrane receptors (i.e., via receptor channel opening, G‐protein mediated interactions, transmembrane receptor signaling, or nuclear receptor signaling) or (ii) intracellular targets (i.e., via internalization through endocytosis, micropinocytosis, phagocytosis, or fusion; or via cargo transfer into cells through gap junctions or nanotubes), especially for therapies that modulate gene transcription or translation [36, 37, 38, 39].

While microenvironment modulation technologies help promote passive targeting of therapies by facilitating their transit across tissue barriers, other technologies can help promote more active targeting of therapies by facilitating cellular interactions. These include: (i) increasing therapy bioavailability (i.e., via PEGylation—the process of covalently attaching polyethylene glycol (PEG) polymer chains to a molecule to enhance its solubility, stability, circulation time, and biocompatibility); (ii) augmenting therapy targeting, binding, internalization, or ability to transfer cargo, either directly (i.e., using priming techniques) [40, 41] or indirectly (i.e., by functionalizing using ligands, uni‐ or bi‐specific monoclonal antibodies, cell penetrating peptides) [42, 43, 44]; and (iii) creating specialized carrier‐platforms that can protect therapies from premature inactivation or degradation by the tissue microenvironment (i.e., nanocarriers, viral, and nonviral vectors) [13, 45]. Newer technologies include creating stimuli‐responsive platforms that can release therapies on local environmental (i.e., tissue hypoxia) or external (i.e., using energy carried within the waves or beams of conventional imaging modalities) cues, for spatial and/or temporal control of therapy delivery [46].

When considering non‐invasive therapies (i.e., thermal, light and radiation therapies), their ability to accurately target, and hence interact with cells, relies heavily on imaging guidance. In addition, the tissues and microenvironments that these therapies need to travel through to reach target cells must be carefully considered, given they can potentially interact, attenuate, or even modify, the therapy. This is best illustrated with stereotactic radiotherapy (i.e., Gamma Knife system), which can deliver radiation therapy in the form of gamma‐rays with 1 mm accuracy using three‐dimensional imaging, but whose delivery must be carefully planned to not damage non‐target cells within the beam path, while concurrently ensuring the beam has enough therapeutic energy to be effective.

### 2.4 Integration of *Precision Delivery* Modules

**FIGURE 3:**
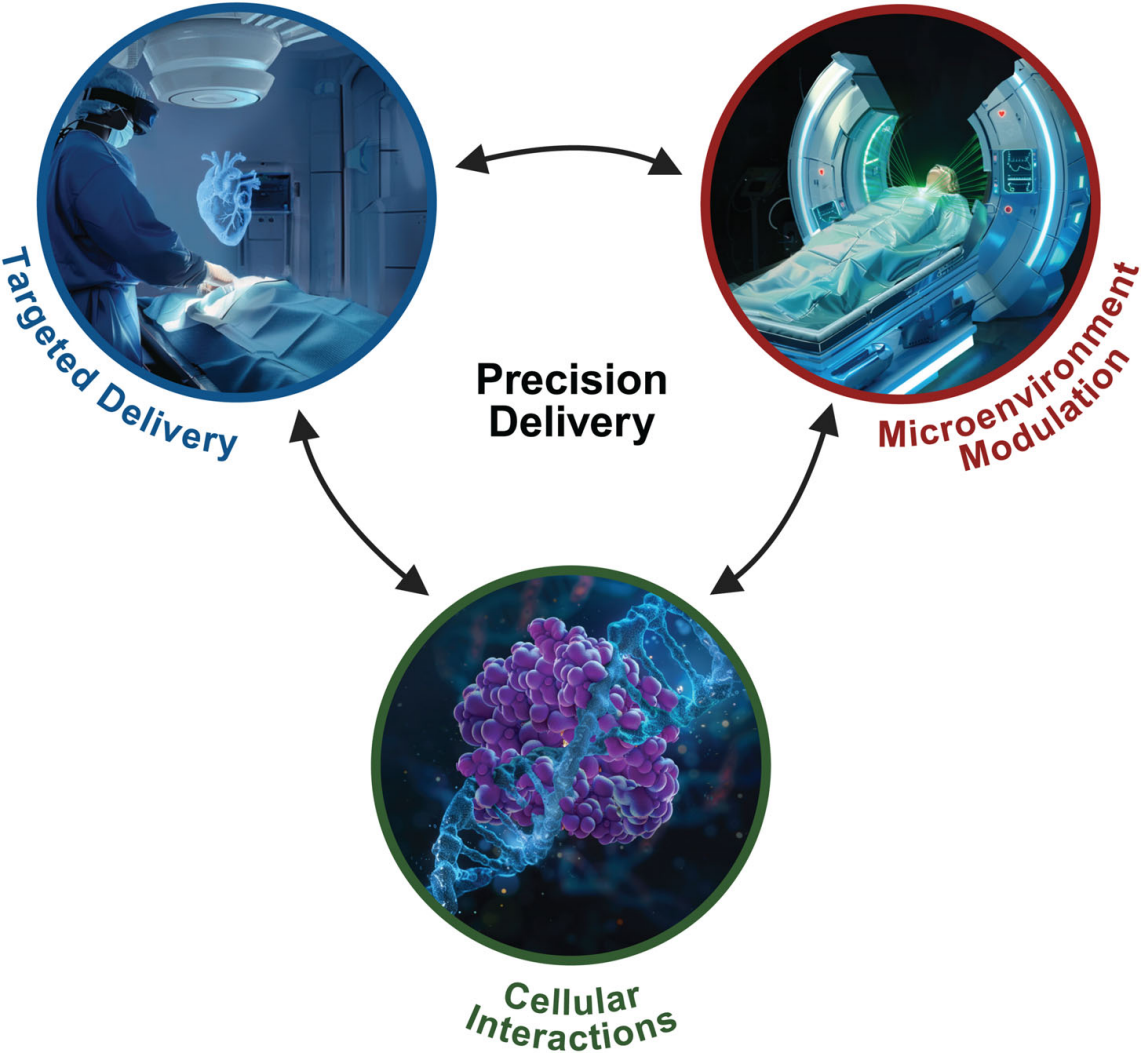
Interactions of the different modules of *Precision Delivery*. *Precision Delivery* consists of three main modules: (i) targeted delivery (delivering therapies directly to specific target locations), (ii) microenvironment modulation (modulating target tissues to make them more responsive to therapies), and (iii) cellular interactions (optimizing therapies to facilitate their interactions with target cells). While each of these modules are independent and can thus be selectively optimized, they can also be combined (i.e., the concurrent use of two or even all three *Precision Delivery* modules) to create synergistic effects to further enhance the effect(s) of *Precision Delivery*. Created with BioRender.com.

When considering existing therapies, new therapies (i.e., gene, cell, cell‐free and immune based therapies), and emerging technologies (i.e., thermal, light, radiation, and electromagnetic field therapies), each of the *Precision Delivery* modules can be independently considered, optimized, and ultimately combined, to ensure more effective translation from the benchtop to the patient's bedside. Over the years, many studies have clearly shown the benefit of employing at least one module of *Precision Delivery* in different disease states. The use of two *Precision Delivery* modules has been less studied, but small clinical studies are starting to emerge showing its benefit—for example, FUS has been used to transiently open the BBB (Module 2) to improve access into the brain of a systemically administered specific antiamyloid monoclonal antibody therapy (aducanumab; Module 3), with evidence showing this approach results in a greater reduction in cerebral amyloid‐beta (Aβ) load in patients with Alzheimer's disease [47]. In another study, targeted IA delivery (Module 1) of a chemotherapy (teniposide) following BBB opening using mannitol (Module 2) was shown to be safe and effective in the treatment of malignant glioma [48]. However, while several preclinical studies clearly demonstrate improved outcomes when using all three *Precision Delivery* modules, clinical studies are currently limited. Of those that exist, the studies are usually based on an extension of an already approved treatment strategy that involves two *Precision Delivery* modules. For example, hepatocellular carcinoma can be effectively treated using transarterial radioembolization (TARE), which uses endovascular techniques (Module 1) to deliver Yttrium‐90 glass beads that accumulate next to cancer cells, blocking their blood supply while also locally delivering high doses of lethal radiation (Module 3). In a recent study, the use of microbubbles that are locally destroyed using ultrasound to prime the tumor microenvironment (Module 2) prior to TARE was shown to be feasible, safe, and have improved patient responses [49]. Hence, if barriers are encountered that prevent the clinical evaluation of an optimized approach that uses all three *Precision Delivery* modules, then a step‐wise approach that builds upon existing foundations that already employ one, or even two, *Precision Delivery* module(s) is recommended (Figure 3).

## 3 Challenges for *Precision Delivery*

### 3.1 Technical Considerations

#### 3.1.1 Technologies to Help Guide Therapy Delivery

As target organs move from superficial to deep locations in the body, imaging plays a central role in guiding both targeted delivery and microenvironment modulation technologies. For example, fluoroscopy and ultrasound are commonly used to guide the placement of medical devices (i.e., needles and catheters) in real‐time, while cross‐sectional imaging, like computerized tomorgraphy (CT) or magnetic resonance imaging (MRI), is the cornerstone for stereotactic guidance of noninvasive technologies (i.e., radiotherapy). Visualizing this information has also evolved from using physical workstations to extended reality technologies (i.e., virtual [VR], augmented [AR], and mixed [MR] reality) that can facilitate real‐time visualization of medical images and information onto patients, which, in turn, will increase the precision of tissue targeting while concurrently reducing procedural time, potential complications, as well as improving operator ergonomics [50]. Furthermore, advances in artificial intelligence (AI) can now extract, segment, and process data within these medical images to identify areas for treatment, as well as create virtual GPS‐like navigation maps that operators can follow to guide devices to specific locations within the body with high accuracy. In addition, advanced predictive simulation modeling (akin to the digital twin concept [51]) can be used to test different *Precision Delivery* strategies. This will be based on (i) patient data, which include imaging that defines the anatomy of a patient, disease location, and disease burden, as well as biodata detailing their physiological status, comorbidities, and even their disease molecular profile; and (ii) available outcome data (i.e., feasibility, efficacy, safety, risk profile) from different approaches or treatment strategies, which will be collected from publications, publicly available databases, or available through electronic medical records that can be efficiently audited through natural language processing techniques [52]. AI algorithms can then integrate this information to create, and select, an optimal delivery strategy tailored to each individual, thereby ensuring the best possible outcome with the lowest risk profile, and hence potential complication rate [53]. Over time, machine learning models will then offer up‐to‐date optimized recommendations based on the latest procedural insights, technological advancements, and overall outcomes that are reported into the model from different centers and industry partners. Furthermore, given the processing power and speed of these AI algorithms, live intraoperative feedback will be possible to help provide instantaneous guidance to the operator, especially through the extended reality space, to ensure optimal delivery approaches are executed. Another growing area of therapy delivery is device tracking, which uses the integration of real‐time sensors into devices that can then provide feedback of surrounding tissue structures (i.e., tissue resistances and even molecular data), as well as positioning within 3D space, thereby determining their exact location within the human body [54, 55]. Given the expansive capacity of AI to process and analyze these large volumes of sensor data, it has also spearheaded transformative advances in medical robotic systems [56, 57, 58].

#### 3.1.2 Monitoring Delivery Outcomes

Our ability to understand how the body responds to therapies is conventionally done by measuring physiological indices, blood markers, or by detecting anatomical changes on conventional imaging; however, these assessments are performed after therapy administration. To enable earlier assessments, newer *Precision Health* monitoring devices (i.e., continuous glucose monitors [CGM]) or molecular/functional imaging approaches (i.e., nuclear medicine imaging) are being integrated into patient follow‐up algorithms. Nevertheless, there are still significant data missing from the point‐of‐therapy‐delivery to the point‐of‐effect, and without this knowledge, it becomes increasingly difficult to understand exactly how the modules of *Precision Delivery* are working and, hence, how they could be further optimized. Hence, several noninvasive in vivo imaging technologies are currently in development, aimed at assessing either the therapy itself (i.e., its viability, biodistribution, activation, and even integration into the target cell), which often requires ex vivo labeling or modification of the therapy, and/or the effect of therapies on target tissues. In the latter case, there has been considerable success in imaging techniques (i.e., with PET and SPECT imaging) using functionalized imaging agents directed at target receptors that are sufficiently upregulated for imaging agents to localize to, and thus generate a detectable signal. However, when these target receptors are limited, or the process being detected is subtle, systemic delivery of imaging agents can encounter similar issues to therapies, ultimately resulting in them not being able to produce enough signal‐to‐noise ratio that can be reliably detected for image generation; in these instances, employing *Precision Delivery* approaches for these imaging agents may have significant benefit. Furthermore, such approaches will be critically important when considering theragnostic agents, especially for radionuclides (i.e., alpha‐emitting nuclides) that could have deleterious off‐target effects if not specifically localized to target tissues [59, 60]. Finally, the timing of implementing different *Precision Delivery* modules needs to be considered; for example, real‐time feedback of biological effects or expression of relevant targets following tissue priming after microenvironment modulation will be critical to guide the optimal time to undertake targeted delivery, or even predict therapy responses that could then help guide potential additional modulation steps to further improve therapy outcome [61].

### 3.2 Clinical Considerations

#### 3.2.1 Patient Variability

While the modules of *Precision Delivery* provide a broad framework to create delivery approaches, patient specific variables should also be considered to create more “personalized” delivery approaches. Hence, in addition to therapy considerations for each patient (i.e., pharmacogenomics, pharmacokinetics and pharmacometabolomics), delivery considerations include: (i) anatomic variations that may preclude, or increase the risk of, certain interventions or technologies; (ii) age‐related changes that can make targeted delivery approaches more challenging (i.e., endovascular delivery: endothelial diseases like atherosclerosis can potentially impact delivery across the vessel wall; endoluminal delivery: the microbiome can modify drug efficacy) [62, 63]; (iii) health and mobility considerations (i.e., specific body positioning, claustrophobia, contrast allergies) that can affect the ability of patients to undertake some procedures, especially if prolonged; (iv) pediatric versus adult patients—delivery can be more challenging in children given their smaller size and the understanding that most children will need general anesthesia, with some studies now showing this may have potential neurodevelopment risks [64].

#### 3.2.2 Standardization and Protocols

Implementation of new technologies often invite challenges due to initial lack of defined standard operating procedures. This disparity can generate reproducibility issues across various sites that then impedes the widespread embracing of such technologies. However, with time and experience, optimized parameters and protocols become established, and as this knowledge is shared through vendors, conferences, publications, or collaborations, it enables more consistent outcomes, which, in turn, promotes wider acceptance and implementation of any new technology. One example to illustrate this point is the use of MRI; initially access to this technology was constrained to a few large centers; however, as this technology became more affordable and imaging protocols became standardized, MRI gradually found its place into more centers and more widely integrated into patient care pathways. Similar implementation flows are to be expected when considering how *Precision Delivery* approaches can be more widely adopted, especially in smaller centers (see “hub‐and‐spoke” model in Section 3.3.1).

For technology applications that have a threshold level effect (i.e., tissue destruction that consistently occurs once a certain amount of energy is deposited), issues with clinical standardization are less evident. However, for effects that occur over a range of input energies (i.e., bioeffects from microenvironment modulation technologies), standardizing approaches becomes challenging, especially when vendors of the same technology use different terminologies, or do not display the exact parameters that are employed. Furthermore, many of these technologies are often preclinically tested by multiple groups using different, and often randomly chosen, parameters; this leads to a complex landscape in which specific bioeffects are reported to occur across large parameter ranges. Hence, to ensure new applications for these novel technologies can be effectively clinically translated, regulatory bodies, societies, and/or foundations are often needed to help create, and implement, standardized protocols. One example is the FUS foundation, which not only provides resources and information for US FDA approved ablative FUS, but has also helped create a central repository to highlight the multiple expanding biomechanical effects of nonablative FUS (i.e., increasing endothelial and ECM barrier permeability, upregulating tissue antigens, cytokines, chemokines and receptors, neuromodulation, etc.) and where they stand in their translational pathway (i.e., preclinical, pilot trials, pivotal trials, US FDA approval, reimbursement) [65]. Through such communities, clinicians, researchers, and industry leaders have been able to come together to create “white papers” to help standardize protocols, as well as access funds for new research and clinical trials to keep pushing forward this technology into new domains.

A final consideration applicable for both targeted delivery and microenvironment modulation is their frequency of implementation. In some circumstances, the repetitive use of certain *Precision Delivery* approaches may be prohibitory (i.e., the need for repetitive sedations/anesthesia or access site risks); under these situations, creative solutions and protocols should be considered, such as using *Precision Delivery* for therapy initiation or rescue (i.e., sub‐optimal or declining responses), with systemic administration then used for maintenance dosing.

#### 3.2.3 Therapy Selection and Modifications

Delivery strategies can be influenced by understanding both the target and mechanism of action of therapies. For example, when treating a diseased segment of bowel, this could be approached from the endoluminal side (via endoscopy) for therapies that preferentially target epithelial cells [66], or from the endovascular side (via the mesenteric circulation) for therapies that preferentially target endothelial cells [14]. The route of administration is also important to consider for certain therapy derivatives; for example, prednisone (a prodrug that the liver converts to its active form, prednisolone) is typically only administered orally in liquid or tablet form, whereas prednisolone (or closely related compounds like methylprednisolone) is given parenterally. Finally, therapy formulations are important to consider based on their absorption, distribution, metabolism, and elimination from different administration routes; for example, lidocaine can be given in sterile isotonic solution (for IV injection), mixed with oils or waxes to produce a semi‐solid cream (for topical applications), incorporated into temperature sensitive adhesive matrices (for transdermal patches), and even suspended in a liquid (for spray applications). Furthermore, new innovative ways are being developed to deliver lidocaine, including incorporating it into liposomes, microneedle array patches, or in ultrasound responsive platforms (i.e., microbubbles or microcapsules made of poly(lactic‐co‐glycolic acid, which is a biodegradable and biocompatible copolymer).

The past decade has also seen significant advances in biological therapies and these need to be carefully considered when using such therapies compared with conventional drug or small molecule therapies, which are highly standardized, reproducibly manufactured, and can be stored and reconstituted in ways which have almost no impact on the therapy. In contrast, cells are living and hence are very sensitive to environmental cues and handling, which can significantly affect their viability, functionality, and overall therapeutic efficacy. This becomes important when cells are given using targeted delivery devices, which can subject them to increased pressures and sheer forces, causing them to become inadvertently activated, or even deactivated/damaged. However, several technologies are in development to mitigate these effects [67], with examples including pressure sensing‐closed loop infusion systems and flow filters to promote laminar flow dynamics during infusions.

### 3.3 Implementation Considerations

#### 3.3.1 Access and Cost

**FIGURE 4:**
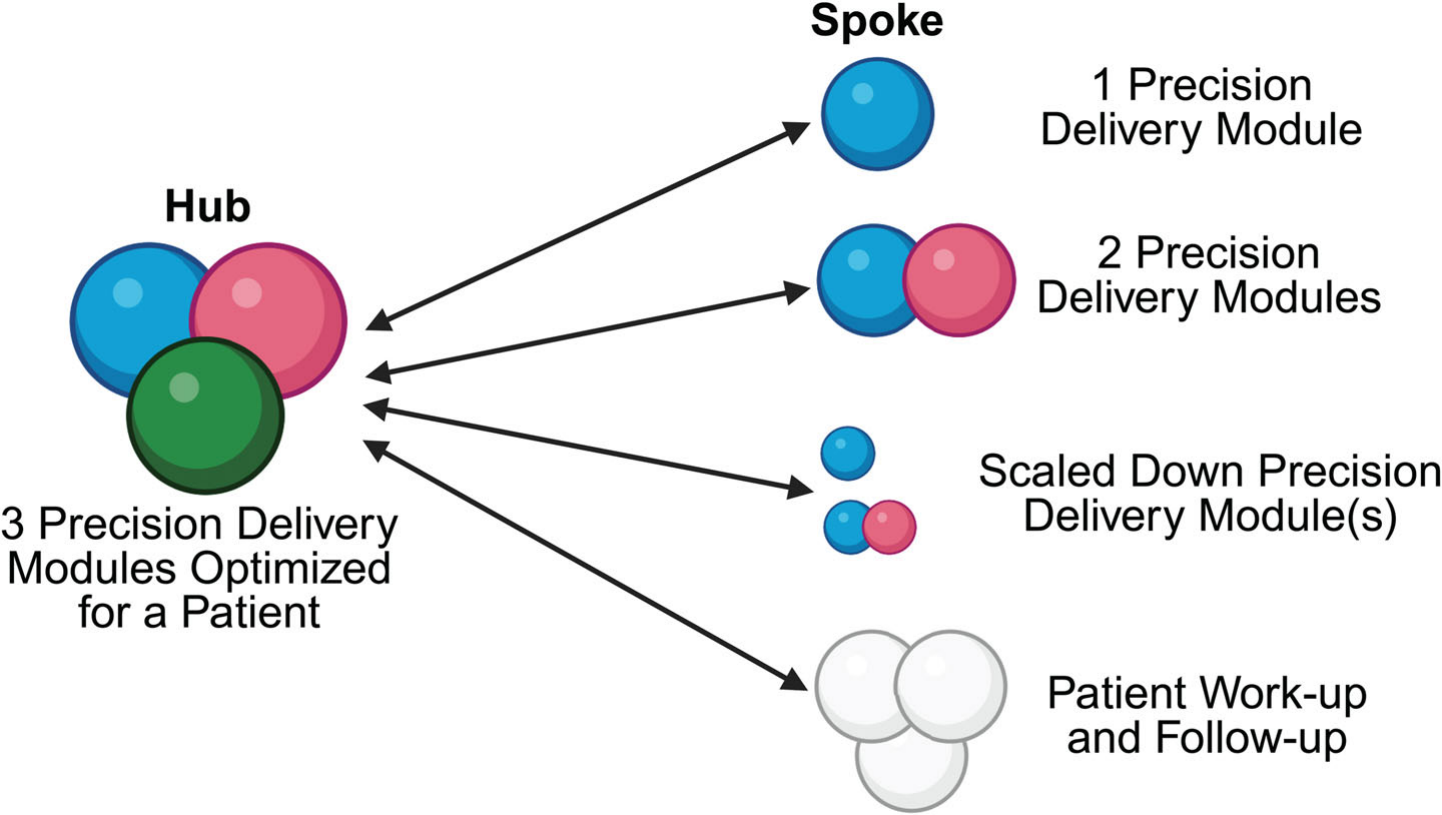
Hub and spoke model for *Precision Delivery*. One potential model to ensure the widespread adoption of *Precision Delivery* in different healthcare systems is to adopt a “hub‐and‐spoke” approach. Here, all modules of *Precision Delivery* can be implemented in academic and larger centers (i.e., the hub), which have sub‐specialty expertise as well as access to specialized infrastructure and expensive equipment. In contrast, smaller centers (i.e., the spokes), will either be able to refer patients into the hubs for *Precision Delivery* approaches, or offer some adaptation of an optimized *Precision Delivery* approach for patients based on their available resources—this can include offering one or even two *Precision Delivery* modules, scaled down *Precision Delivery* modules, or even just working up patients before, and following them after, their transfer into the hubs whey they can receive a fully optimized and individualized *Precision Delivery* approach. For such a model to work, the overarching governance structure relies on extensive collaborations, excellent communication between healthcare providers, seamless sharing of protected healthcare information, and a robust transfer‐infrastructure into and out of the hub, to ensure patients can all benefit from *Precision Delivery* approaches, regardless of their location or direct access to specific centers. Created with BioRender.com.

In general, specific operator skills are required to perform targeted delivery techniques, while access to specialized and expensive equipment is often required for microenvironment modulation approaches; both of which are readily available through interventional and surgical specialties in larger hospitals and academic centers. However, in smaller community hospitals, especially in low‐resource areas, implementing *Precision Delivery* approaches can be challenging due to limited infrastructure and technical experience, as well as cost constraints. To address this, a “hub‐and‐spoke” model has proven successful in many other areas of medicine [68], which when applied to *Precision Delivery*, could mean that more advanced technologies and expertise will be concentrated in the central hubs, while the spokes would focus more on patient work up and follow‐up with transfer into the hubs for streamlined therapy delivery. For some patients who may not be able to travel to hubs due to physical, family, or financial constraints, more advanced care coordination will likely be required, including offering select, or scaled down, *Precision Delivery* modules based on available resources to ensure that patients at these spoke locations receive some benefit compared with conventional delivery approaches (Figure 4). Furthermore, for this framework to work, accessibility to *Precision Delivery* modules at the hubs will have to accommodate an increased throughflow; in principle, this can be accomplished by educating and training nonprocedural physicians, and even nonphysician medical providers (i.e., physician assistants), to perform the smaller and lower risk delivery procedures, with more technically challenging procedures that use complex technologies triaged to those with more subspecialized skillsets. However, the overall implementation of such models is always associated with challenges in private health care systems, where insurance coverage may dictate whether a patient can access a hub or receive coverage for an optimized *Precision Delivery* approach.

#### 3.3.2 Regulatory Compliance

For new therapies, *Precision Delivery* strategies can be prospectively integrated into new clinical trial workflows for subsequent evaluation and approval. In contrast, the use of such strategies for established therapies can come with challenges, especially as their approval will have been granted on prior clinical trial outcomes, in which dosing and pharmacokinetics will have been assessed following systemic administration, thereby potentially making these datasets not applicable for targeted delivery approaches. In these circumstances, the ability to integrate emerging technologies and *Precision Delivery* strategies into established delivery workflows and protocols needs to be done with care to ensure: (i) the new delivery approach falls within acceptable standards of patient care; (ii) patients and clinical teams understand the additional benefit these new approaches offer relative to their risk profile; (iii) therapies are compatible with the new technology; and (iv) any proposed targeted delivery route is approved within the instructions for use for a therapy. If these criteria are not met, consultation and guidance from the therapy and technology companies will be needed, followed by local Institutional Review Board approval to conduct an investigational study to demonstrate the safety and efficacy of the delivery approach with the chosen therapy, especially since potential dose reduction strategies should be considered to mitigate adverse effects arising from high local concentrations of a therapy.

#### 3.3.3 Patient Consent and Data Privacy

For *Precision Delivery* strategies adapted from existing techniques that are currently used in clinical practice, the benefits and risks can be easily explained and contextualized for informed patient consent. (That is, lumbar puncture has traditionally been used as a safe and easily performed procedure for cerebrospinal fluid sampling, but recently it is now being used as an access route for targeted therapy delivery into the central nervous system—one example is spinraza/nusinersen for the treatment of spinal muscular atrophy). However, for more invasive approaches that use novel techniques or technologies that have a higher risk profile, it will be important to clearly explain this to patients to ensure they are fully informed. In situations where patients do not feel comfortable with an approach, specific *Precision Delivery* modules may be able to be either removed or substituted based on supporting evidence and within the limitations set by any clinical trial. Furthermore, technologies that rely on extensive data input and collection have the potential to introduce new risks to patient privacy, especially when these datasets are shared to improve either the technology itself or drive *Precision Delivery* AI algorithms. Hence, appropriate measures and safeguards need to be set in place from the outset, including using de‐identified data, employing data security protocols, being compliant with HIPPA guidelines, as well as being transparent to patients about how their data will be used to ensure they can provide their informed consent and maintain their trust with the healthcare provider.

## 4 The Future of *Precision Delivery*

For *Precision Delivery* to reach its full potential, thoughtful preclinical testing needs to be undertaken in clinically appropriate translational models that not only have a similar anatomy to humans but also exhibit diseases that share the same molecular pathways observed in patients. These studies should also focus on generating scientific evidence to support an optimal approach for clinical translation, demonstrating the benefits that each *Precision Delivery* module has in isolation, as well as the synergistic effects when they are combined. Close collaboration and early engagement with regulatory bodies (such as the US FDA) is strongly recommended to ensure that the correct and appropriate datasets are collected to support an optimized delivery approach, thus facilitating a more streamlined translation into clinical trials. In late‐stage preclinical testing in larger animal models, the equipment used should be as close as possible to versions that will be clinically translated to allow for iterative feedback on the setup, protocols (i.e., settings and algorithms), and use (i.e., interactions with the user and patient) before testing in human patients. Furthermore, given some devices are driven by complex and sophisticated engineering technologies, it will be important for them to have simplified interfaces, moving away from multiple dials and knobs, to one‐touch preprogrammed buttons that can execute an automated protocol; this will enable more widespread use of technologies across different centers and operators, while concurrently reducing the risk for any intraprocedural errors. The algorithms used to create these protocols will be based on preclinical datasets and will work by dynamically adjusting intraprocedural parameters to ensure the optimal delivery of therapies, while operating within a predefined safe range to minimize potential harm/adverse effects.

Despite the insights and technological advances contributing to each *Precision Delivery* module, majority of clinical data are from small single‐center early phase (i.e., I and II) studies; while these often show good safety and efficacy data supporting various delivery approaches, there is still a lack of robust long‐term data (especially survival data for cancer studies) from larger prospective, randomized, controlled, multicenter phase III clinical trials, that directly compare these novel delivery approaches with conventional systemic delivery. This is further complicated by the fact that many of these approaches also employ concomitant conventional or newer targeted (i.e., immunotherapies in oncology) therapies in the background; as such, it is often hard to match patients with similar disease burden and molecular characteristics. In addition, care must be taken when drawing conclusions from studies in which microenvironment modulation was essential for a therapy to reach target cells but which was not performed, irrespective of the delivery approach. Furthermore, given that more than one module will likely be needed to fully realize *Precision Delivery* for many diseases, either robust preclinical data will need to be generated to validate an optimized delivery approach, or each *Precision Delivery* module will need to be assessed in double/triple‐intervention trials, such that they can be evaluated as an independent variable in isolation, or in combination, relative to a conventional treatment arm. Given the complexity of undertaking such trials, consortiums will likely need to be formed across multiple centers to facilitate protocol standardization, patient recruitment, as well as broader population sampling to ensure reduce any bias in the data. To address concerns related to these exploratory trials not adequately informing clinical practice or policy decisions in the real‐world, given they are usually optimized to determine efficacy, pragmatic trials should also be considered to assess how complex interventions, consisting of several interacting components, can be assessed for meaningful outcomes relative to acceptable standards of care [69].

New and exciting areas for therapy delivery include the fetus, children/pediatrics, and the lymphatic system; all of which have seen recent significant developments in delivery approaches due to technological advancements in both imaging (that can be used for interventional guidance) and equipment. Other exciting developments include delivery of neuromodulatory implantable chips (i.e., Nerualink) that act as tissue‐computer interfaces to detect and process neuronal action potentials to wirelessly control external devices. Wearable devices are also set to play a key role in understanding and assessing the effectiveness of therapies by monitoring their responses following *Precision Delivery* approaches (i.e., using a CGM to assess the function of transplanted islets following their endovascular delivery into the liver) or even help guide treatment timing based on physiological or molecular changes that can be detected with these devices. Finally, *Precision Delivery* solutions are increasingly being appreciated to be essential for emerging exciting therapies such as: (i) CRISPR–Cas9 (clustered regularly interspaced short palindromic repeats‐associated protein 9), which is a powerful gene editing technology that can modify DNA at specific locations to insert, delete, or alter the DNA sequence to modify and correct abnormal genes with the hope of curing certain diseases, but which have encountered poor editing efficiency, off‐target effects, and immunogenicity issues when translated into humans; and (ii) nanotechnologies that have shown significant preclinical potential, with theranostic, stimuli‐responsive, microenvironment modulating, and multimodal nanoprobes [70], but which have also not been easily translated into patients due to biodistribution issues (i.e., predominant uptake in the reticuloendothelial system following systemic administration) and off‐target toxicity issues.

## 5 Conclusion

Although systemic administration of some therapies may be appropriate, this delivery choice should not be the default route, nor the one used based solely on convenience. Instead, therapy delivery should be considered in the organized framework of *Precision Delivery* to ensure the correct module(s) is(are) used and optimized for each individual patient. As AI integrates into *Precision Delivery*, to process the outcome and safety data from the different modules relative to each patient and disease state, it will catalyze the development of predictive models to help physicians choose the most appropriate patient‐specific delivery approach for a certain therapy, as well as catalyze innovation to create solutions to address new unmet delivery needs or existing shortcomings. Hence, integrating *Precision Delivery* into Precision Medicine in the coming decade has the potential to ensure that each patient will be able to get the right therapy, at the right place, at the right time.

## Author Contributions

The manuscript was conceptualized, designed, and written by Dr Avnesh S. Thakor.

## Ethics Statement

The author has nothing to report.

## Conflicts of Interest

A. S. T. is a cofounder and holds stock options for Teal Health and is on the Scientific Advisory Board, received grants, or is a consultant for RespondHealth Inc, Cellular Vehicles Inc, Nephrogen Inc, ReThink64 Inc, AlloTRx Inc, Inari Inc, and Genentech Inc.

## Data Availability

The author has nothing to report.
